# Effects of a cognitive-based intervention program using social robot PIO on cognitive function, depression, loneliness, and quality of life of older adults living alone

**DOI:** 10.3389/fpubh.2023.1097485

**Published:** 2023-02-06

**Authors:** JunSeo Lim

**Affiliations:** The Research Institute of Nursing Science, College of Nursing, Seoul National University, Seoul, Republic of Korea

**Keywords:** social robot, cognitive function, depression, loneliness, quality of life, older adults

## Abstract

**Objective:**

Social robot interventions are being implemented to reduce cognitive decline, depression, and loneliness among older adults. However, the types, functions, and programs of effective social robots have not yet been confirmed. This study investigated whether a social robot intervention is effective in improving cognitive function, depression, loneliness, and quality of life in older adults living alone.

**Methods:**

This study used a non-equivalent control group pre-test–post-test design. It was conducted twice a week, with each session lasting 50 mi; twelve sessions were conducted over 6 weeks. This study was conducted at three senior welfare centers in Korea. In each group, 10 or fewer participants used the PIO social robot. The total participants included 64 people in the experimental (*n* = 31) and control groups (*n* = 33), and consisted of older people over 65 years of age living alone.

**Results:**

There was a statistically significant difference in the pre-post values for cognitive function (*z* = 5.21, *p* < 0.001), depression (*z* = −2.99, *p* = 0.003), and loneliness (*t* = −4.27, *p* < 0.001) in the experimental and control groups. However, there was no statistically significant difference for quality of life (*z* = 1.84, *p* = 0.066).

**Conclusions:**

It was confirmed that a cognitive intervention program using the social robot PIO can improve cognitive function and reduce depression and loneliness in older adults living alone.

## 1. Introduction

The global population aged over 65 years is expected to increase from 9.3% in 2020 to approximately 16% in 2050 (1). The population of those aged 65 years and above in Korea was 15.7% in 2020; this population of older adults belongs to an aging society, among which the proportion of those living alone is 19.6% (2). Older adults who live alone experience deterioration in their health owing to aging, economic poverty, dependence due to reduced income, loneliness due to social and psychological conflicts, severance of human relationships, and helplessness due to loss of social roles (3). In addition, older people living alone are more vulnerable compared to those living together concerning physical health, mental health, and quality of life (QoL) due to a lack of social cohesion through family relationships (4), social isolation, and reduced social support (5).

The decline in cognitive function due to aging reduces an individual's ability to adapt and causes emotional problems such as depression and anxiety, making it difficult to form interpersonal relationships that lead to poor QoL (6). Depression affects QoL in the older adults (7, 8), and living alone is a contributing factor to depression (5). Loneliness not only causes suffering in older adults by itself, but also indicates mental disorders such as depression, dementia, suicide, and physical diseases such as hypertension, heart disease, and diabetes (9, 10). Various non-pharmacological interventions are being tried in various ways to prevent cognitive decline and increase cognitive function in the older people. A cognition-based intervention is a comprehensive and complex approach aimed at improving cognitive function, cognitive stimulation, cognitive training, and cognitive rehabilitation (11). Computer-assisted cognitive rehabilitation (CACR) research has been increasing recently. In CACR, the group that received cognitive training using only a computer program saw a significant effect on cognitive function and memory but no effect on emotions or emotional variables (12). Various intervention studies using social robots are being attempted worldwide to alleviate the burden of care for older people amid changes in the family structure. The PARO robot is an emotional support robot that responds to touch, light, sound, and temperature through bodily sensors (13). A meta-analysis of the PARO robot showed that the effects were inconsistent on variables, such as cognitive function, problematic behavior, depression, and loneliness (14, 15). As a result of a cognitive intervention using Sil-Bot, the social robot, thinning of the anterior cingulate cortex was reduced, and age-related structural brain changes could be alleviated (16). Social robot programs for older people are mostly applied by substituting animal-assisted therapy (AAT) programs (17) with them or by placing social robots in centers and through facilitating interaction with them. As per past research, traditional cognitive training, such as stimulating the senses, was performed (18–20), and the study variables were mainly emotional. However, it was difficult to find studies that applied a CACR program to social robots to improve participants' cognitive functions and confirm emotional variables through interactions between social robots and participants.

Therefore, this study aimed to improve the cognitive function of older adults living alone by using the social robot PIO, to which the CACR program was applied. In addition, this study aimed to confirm the effect of reducing depression and loneliness and improving QoL through the interaction of the social robot PIO.

## 2. Methods

### 2.1. Study design

This was a non-equivalent control group pre-test–post-test design.

### 2.2. Participants

Participants were recruited from November 22, 2021 to January 5, 2022, at three senior welfare centers in Seoul, Korea. Forty-one people in the experimental group and 39 in the control group expressed their intentions to participate. Among them, five from the experimental group and three from the control group were excluded because they did not meet the selection criteria; 36 individuals each from the experimental and control groups finally participated in the study. During the study, three of 36 experimental group subjects dropped out due to health problems, two in the program refused to participate, and three out of 36 in the control group did not participate in the investigation due to COVID-19 infections. All participants adhered to the program schedule and attended all the sessions. There were no dropouts during the course of the intervention. The recruitment criteria for participants were as follows: (1) 65 years of age or older, (2) older adults living alone, (3) able to communicate in Korean, and (4) no history of severe mental illness, such as schizophrenia or delusional disorder. Exclusion criteria were: (1) older adults who were in the new cognitive intervention program at the time of the study, (2) history of psychiatric drug use within the last 4 weeks, and (3) having difficulty using their hands. Participants who wished to take part in the program were assigned to the experimental group first, and the control group was selected using convenience sampling by matching participants with those in the experimental group in terms of age, gender, education, and basic livelihood security recipient status. The final data were collected from 64 participants, who were divided into the experimental (*n* = 31) and control groups (*n* = 33). The sample size was calculated using G^*^power (3.1.9.6), with a significance level of 0.05, a large effect size (0.80), and a power of 0.85. According to the preliminary analysis, at least 30 people were required for each group, and an additional 20% were recruited considering the dropout rate.

### 2.3. Intervention

**Figure 1:**
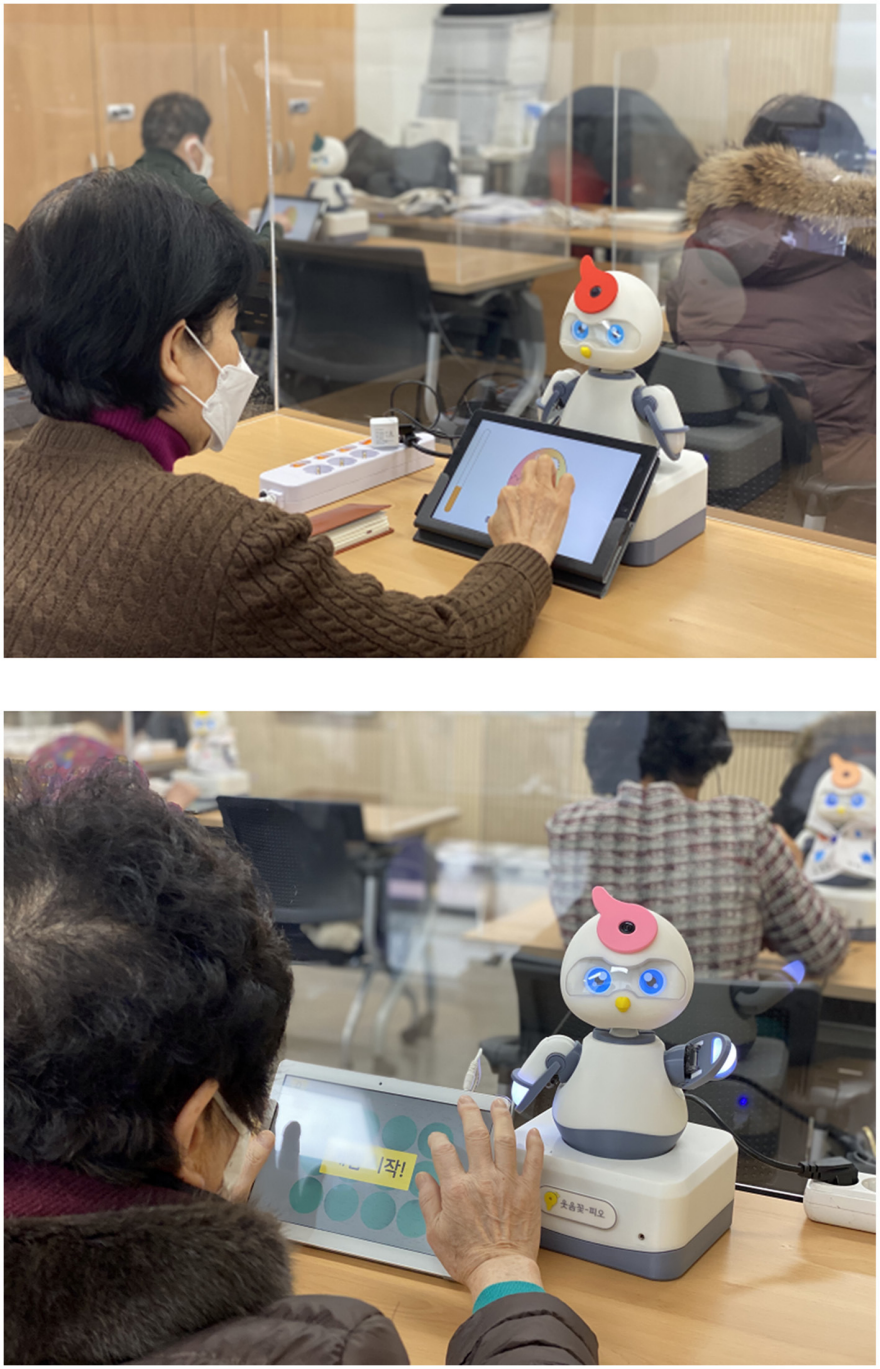
Participants conducting PIO program.

The intervention in this study was conducted from January 10 to March 2, 2022. During the study period, participants continued to participate in usual care (music, art programs, etc.) that they had previously enrolled in at the senior welfare center, and did not stop or participate in other programs. The experimental group participated in this study's program, and the control group did not. This program was conducted twice a week, 50 min per session, for 6 weeks. Each group of 10 or fewer participants underwent PIO. For smooth progress, two research assistants were used. The program, which consisted of 12 sessions, was structured by a storytelling of the process behind the parrot-shaped robot PIO hatching from an egg and growing into an adult robot. Each session started with the themes of “meeting with the social robot PIO” and “recalling the previous program.” In addition, gymnastics was performed with the social robot PIO to relieve tension and improve intimacy with the robot. Before the 6^th^ session, “Teaching movements and gymnastics,” gymnastics was performed while watching a video, and after the 6^th^ session, gymnastics was performed while watching the movements of the social robot PIO. The program lasted about 30 min according to the contents of each session. The ending involved “Expressing your impression about the program” and “Saying goodbye to social robot PIO” (Table 1). Participants were configured to naturally stimulate cognition and emotion in the process of PIO programs (Figure 1).

**Table 1:** Contents of PIO program.

	**Contents**	**Activities**	**Therapeutic factors**
1	Hatching an egg	Program introduction	Emotion
		Stretching exercise	Cognition, exercise
		Shake eggs to hatch	Cognition, exercise
2	Making up baby PIO	Stretching exercise	Cognition, exercise
		Making up baby Social Robot	Cognition, art, emotion
		Nest decoration for baby Social Robot	Cognition, art, emotion
3	Feeding and putting baby PIO to sleep	Stretching exercise	Cognition, exercise
		Feeding milk with formula	Cognition, emotion
		Put a crying baby Social Robot to sleep	Cognition, emotion
4	Making clothes for PIO	Stretching exercise	Cognition, exercise
		Make clothes	Cognition, art, emotion
5	Teaching baby PIO to speak	Stretching exercises	Cognition, exercise
		Words for the situations	Cognition, emotion
		Searching words	Cognition, emotion
6	Teaching movements and gymnastics	Stretching exercises	Cognition, exercise
		Teach movements	Cognition, exercise
		Doing gymnastics with Social Robot	Cognition, exercise
7	Catching caterpillars	Doing gymnastics with Social Robot	Cognition, exercise
		Catching caterpillars	Cognition, exercise
8	Catching giant caterpillars	Doing gymnastics with Social Robot	Cognition, exercise
		Catching a moving giant caterpillar	Cognition, emotion
9	Teaching colors	Doing gymnastics with Social Robot	Cognition, exercise
		Teaching color words	Cognition, emotion
		Coloring caterpillars	Cognition
		Catching caterpillars	Cognition
10	Shopping with PIO	Doing gymnastics with Social Robot	Cognition, exercise
		Shopping at the supermarket	Cognition
		Order the scenes	Cognition
		Remember locations	Cognition
11	Shopping with PIO	Doing gymnastics with Social Robot	Cognition, exercise
		Clapping with Social Robot	Cognition, music
		Drawing portrait	Cognition, art, emotion
12	Farewell	Doing gymnastics with Social Robot	Cognition, exercise
		Recall the entire programs	Cognition, emotion
		Sharing your feelings	Emotion

### 2.4. Social robot PIO

**Figure 2:**
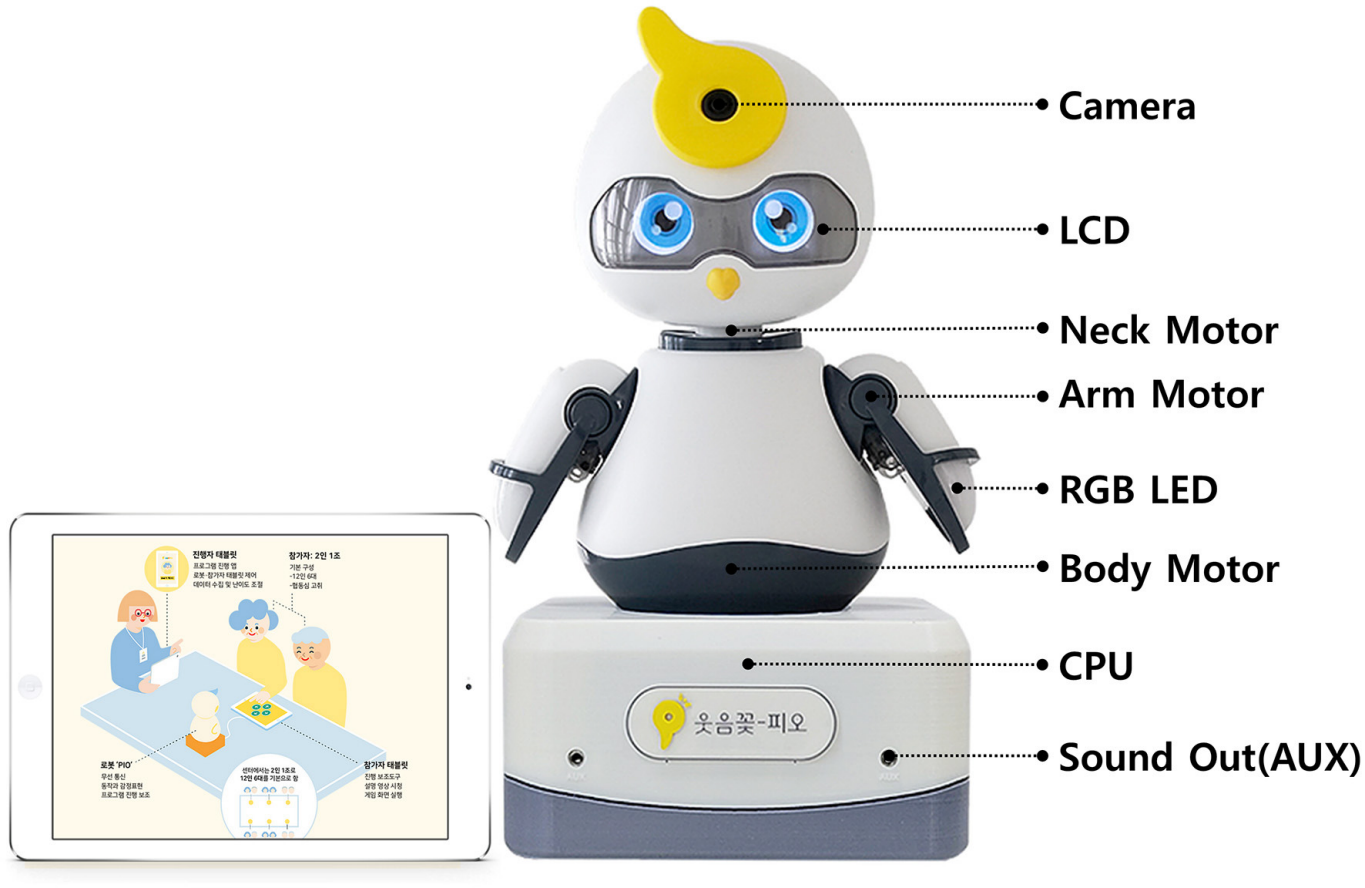
Social robot PIO (Version 2.0).

The social robot PIO is a parrot-shaped robot developed by Why Dots for cognitive-emotional programs with older people and older adults with dementia. The social robot PIO expresses various emotions through the eyes of an LCD display, and it is possible to implement structured conversations and various movements according to the program composition when performing a program with subjects. The size of the social robot was 200 ×170 ×325 mm, and its weight was approximately 2 kg (Figure 2).

### 2.5. Instruments

#### 2.5.1. K-MMSE~2:SV

The Korean-Mini Mental State Examination, 2nd Edition (K-MMSE~ 2): SV (Standard Version) by Kang et al. (21), which translated the 2010 revised Mini-Mental State Examination (MMSE) developed by Folstein et al. (22) into Korean, was used. The K-MMSE~2:SV comprises seven subscales in the following order: registration, orientation to time, orientation to place, recall, attention and calculation, language, and drawing. At the time of instrument development, Cronbach's α was 0.69 (21). In this study, Cronbach's α was 0.73.

#### 2.5.2. Depression

The Geriatric Depression Scale Short Form: Korean Version (GDSSF-K), a tool developed by Sheikh and Yesavage (23), which was translated and modified by Kee (24), was used to measure depression. The GDSSF-K consists of 15 items and “yes” or “no” answers to each question. The higher the score, the greater the depression. At the time of development, the Cronbach's α was 0.88. In this study, the Cronbach's α was 0.79.

#### 2.5.3. Loneliness

The Revised UCLA Loneliness Scale (RULS), developed by Russel et al. (25), was used to measure loneliness; the scale was translated into Korean by Kim (26). It consists of 20 items on a four-point Likert scale, and the higher the score, the greater the degree of loneliness. At the time of tool development, Cronbach's α was 0.93. In this study, Cronbach's α was 0.87.

#### 2.5.4. QoL

QoL was measured using the EQ-5D-3L (Euro Quality of life-5Dimension-3Level) tool developed by the EuroQol group. The EQ-5D-3L consists of five domains: mobility, self-care, usual activities, pain/discomfort, anxiety/depression. In this study, the QoL tariff by Lee et al. (27) was used for calculation. In the sub-domain, the raw score was reverse-coded, with a higher score indicating a higher QoL. At the time of instrument development, Cronbach's α was 0.64 (28). In this study, the Cronbach's α was 0.61.

### 2.6. Statistical analysis

The data collected were analyzed using STATA 17. Descriptive statistics were used to demonstrate the demographic characteristics. The homogeneity between groups was analyzed using the *t*-test, χ2 test, and Fisher's exact test. Tests of major variables to confirm the effectiveness of the program were analyzed with an independent *t*-test for normal distribution and Mann-Whitney U test for alternative distribution. The level of significance was set at *P* < 0.05.

## 3. Results

### 3.1. General characteristics

Demographic characteristics and health information of the participants are summarized in Table 2. In the experimental group, there were 29 (93.5%) females and 2 (6.5%) males; the control group comprised 28 (84.8%) females and 5 (15.2%) males. Concerning age, 11 (35.5%) were 70–74 years old in the experimental group; 11 (33.3%) were 75–79 years old in the control group. Regarding level of education, the majority of elementary school graduates were 9 (29.0%) in the experimental group and 17 (51.5%) in the control group. Regarding marital status, bereavement was common in 18 (58.1%) and 24 (72.7 %) patients in the experimental and control groups, respectively. Regarding the presence or absence of children, there were 29 (93.5%) in the experimental group and 28 (84.8%) in the control group. Regarding the presence or absence of companion animals, 28 (90.3%) participants in the experimental group and 31 (93.9%) in the control group did not have companion animals. Regarding religion, Buddhism was the most common, with 14 (45.2%) participants in the experimental group, and Christianity was the most common, with 12 (36.4%) in the control group. Regarding religion, Buddhism was the most common, with 14 (45.2%) participants in the experimental group, and Christianity was the most common, with 12 (36.4%) in the control group. There were 10 (32.3%) and 18 (54.6%) patients in the experimental and control groups, respectively. Twenty-five (80.6%) patients in the experimental group and 26 (78.8%) in the control group had no physical disabilities. Twenty-four (77.4%) patients in the experimental group and 23 (69.7%) in the control group did not have a basic livelihood.

**Table 2:** Homogeneity test of general characteristics (*N* = 64).

**Characteristics**	**Categories**	**Exp. (*n =* 31)**	**Con. (*n =* 33)**	**χ^2^**	***p***
		***n*** **(%)**	***n*** **(%)**		
Gender	Male	2 (6.5)	5 (15.2)	1.24^†^	0.428
	Female	29 (93.5)	28 (84.8)		
Age	65~69	5 (16.1)	1 (3.0)	6.17^†^	0.206
	70~74	11 (35.5)	7 (21.2)		
	75~79	6 (19.4)	11 (33.3)		
	80~84	7 (22.6)	10 (30.3)		
	85~89	2 (6.5)	4 (12.1)		
Education level	No education	4 (12.9)	4 (12.1)	8.54^†^	0.074
	Elementary	9 (29.0)	17 (51.5)		
	Middle	7 (22.6)	4 (12.1)		
	High	4 (12.9)	7 (21.2)		
	College	7 (22.6)	1 (3.0)		
Married	Single	0 (0.0)	1 (3.0)	3.08^†^	0.358
	Separation	9 (29.0)	5 (15.2)		
	Divorce	4 (12.9)	3 (9.1)		
	Bereavement	18 (58.1)	24 (72.7)		
Children	Yes	29 (93.5)	28 (84.8)	1.24^†^	0.428
	No	2 (6.5)	5 (15.2)		
Pet	Yes	3 (9.7)	2 (6.1)	0.29^†^	0.667
	No	28 (90.3)	31 (93.9)		
Religion	Buddhism	14 (45.2)	7 (21.2)	4.65	0.199
	Christian	6 (19.4)	12 (36.4)		
	Catholic	5 (16.1)	6 (18.2)		
	None	6 (19.4)	8 (24.2)		
Number of chronic diseases	0	1 (3.2)	1 (3.0)	3.29^†^	0.14
	1	20 (64.5)	14 (42.4)		
	≥2	10 (32.3)	18 (54.6)		
Physical disability	Yes	6 (19.4)	7 (21.2)	0.03	0.854
	No	25 (80.6)	26 (78.8)		
Basic livelihood security recipient	None	24 (77.4)	23 (69.7)	0.65^†^	0.821
	Recipient	5 (16.1)	8 (24.2)		
	The secondary lower income family	2 (6.5)	2 (6.1)		

Con., Control group; Exp., Experimental group;

† Fisher's exact test.

### 3.2. Effects of PIO interventions on cognition function

For the cognition function variable, the difference between the pre-post scores of the experimental and control groups were statistically significant (*z* = 5.21, *p* = 0.001). The experimental group increased by 3.00 ± 2.73, and the median (IQR) increased to 2 (3), the control group decreased by −0.61 ± 1.78, and the median value (IQR) decreased to −1 (3). On the subscale, location orientation (*z* = 3.41, *p* < 0.001), attention and counting (*z* = 3.71, *p* < 0.001), and language (*z* = 2.62, *p* = 0.009) increased significantly, but registration (*z* = −0.53, *p* = 0.595), temporal orientation (*z* = 1.70, *p* = 0.089), memory recall (*z* = 1.66, *p* = 0.097), and drawing (*z* = 0.92, *p* = 0.357) did not show statistically significant differences (Table 3).

**Table 3:** Comparison of cognition function (*N* = 64).

**Variables**	**Group**	**Pre**		**Post**		**Post-Pre**		***z***	***p***
**Cognition function**		**M** ±**SD**	**Median (IQR)**	**M** ±**SD**	**Median (IQR)**	**M** ±**SD**	**Median (IQR)**		
Total	Exp. (*n =* 31)	25.87 ± 3.37	27 (5)	28.87 ± 1.52	29 (2)	3.00 ± 2.73	2 (3)	5.21	< 0.001
	Con. (*n =* 33)	25.91 ± 3.21	26 (5)	25.30 ± 3.36	26 (6)	−0.61 ± 1.78	−1 (3)		
Registration	Exp.	2.97 ± 0.18	3 (0)	3.00 ± 0.00	3 (0)	0.03 ± 0.18	0 (0)	−0.53	0.595
	Con.	2.85 ± 0.44	3 (0)	2.91 ± 0.38	3 (0)	0.06 ± 0.24	0 (0)		
Orientation to Time	Exp.	4.74 ± 0.63	5 (0)	5.00 ± 0.00	5 (0)	0.26 ± 0.63	0 (0)	1.7	0.089
	Con.	4.64 ± 0.60	5 (1)	4.52 ± 0.87	5 (1)	−0.12 ± 0.74	0 (0)		
Orientation to place	Exp.	4.42 ± 0.81	5 (1)	5.00 ± 0.00	5 (0)	0.58 ± 0.81	0 (1)	3.41	< 0.001
	Con.	4.88 ± 0.33	5 (0)	4.94 ± 0.24	5 (0)	0.06 ± 0.24	0 (0)		
Recall	Exp.	1.90 ± 1.01	2 (2)	2.48 ± 0.81	3 (1)	0.58 ± 1.06	0 (1)	1.66	0.097
	Con.	2.06 ± 0.97	2 (2)	2.18 ± 0.95	2 (1)	0.12 ± 0.89	0 (1)		
Attention and calculation	Exp.	3.71 ± 1.55	4 (2)	4.58 ± 0.85	5 (1)	0.87 ± 1.52	0 (2)	3.71	< 0.001
	Con.	3.42 ± 1.68	2 (3)	2.67 ± 1.69	2 (3)	−0.76 ± 1.42	0 (1)		
Language	Exp.	7.35 ± 0.84	8 (1)	7.97 ± 0.18	8 (0)	0.61 ± 0.80	0 (1)	2.62	0.009
	Con.	7.27 ± 0.76	7 (1)	7.33 ± 0.85	8 (1)	0.06 ± 0.66	0 (0)		
Drawing	Exp.	0.77 ± 0.43	1 (0)	0.84 ± 0.37	1 (0)	0.06 ± 0.51	0 (0)	0.2	0.357
	Con.	0.79 ± 0.42	1 (0)	0.76 ± 0.44	1 (0)	−0.03 ± 0.31	0 (0)		

Con., Control group; Exp., Experimental group.

### 3.3. Effects of PIO interventions on depression, loneliness, and QoL

For the depressive variable, the difference between the pre-post scores of the experimental and control groups were statistically significant (*z* = −2.99, *p* = 0.003). The experimental group decreased by −2.42 ± 3.36, and the median (IQR) decreased to −2 (3), the control group increased by 0.03 ± 2.82, and the median (IQR) was 0 (3). For the loneliness variable, the difference between the pre-post scores of the experimental and control groups was statistically significant (*t* = −4.27, *p* < 0.001). The experimental group decreased by −11.26 ± 9.34, and the median (IQR) decreased to −12 (14), the control group decreased by −0.55 ± 10.64, and the median (IQR) was 1 (16). Regarding QoL variables, the difference between the pre-post scores of the experimental and control groups was not statistically significant (*z* = 1.84, *p* = 0.066). The experimental group increased by 0.07 ± 0.12, and the median (IQR) increased to 0.09 (0.14); the control group increased by 0.03 ± 0.10, and the median (IQR) was 0 (0.96). On the subscale, mobility (*z* = 3.21, *p* < 0.001) increased significantly, but self-care (*z* = 1.03, *p* = 0.302), usual activities (*z* = 1.02, *p* = 0.310), and anxiety/depression (*z* = 1.85, *p* = 0.064) did not show any statistically significant difference. Pain/discomfort (*z* = −0.63, *p* = 0.530) decreased, but the difference was not statistically significant (see Table 4).

**Table 4:** Comparison of depression, loneliness and QoL (*N* = 64).

**Variables**	**Group**	**Pre**		**Post**		**Post-Pre**		**t^†^ *or z***	***p***
		**M ±SD**	**Median (IQR)**	**M ±SD**	**Median (IQR)**	**M ±SD**	**Median (IQR)**		
Depression	Exp. (*n =* 31)	20.35 ± 3.45	20 (7)	17.94 ± 3.40	17 (5)	−2.42 ± 3.36	−2 (3)	−2.99	0.003
	Con. (*n =* 33)	21.30 ± 3.63	21 (5)	21.33 ± 3.80	22 (5)	0.03 ± 2.82	0 (3)		
Loneliness^†^	Exp. (*n =* 31)	42.45 ± 11.69	42 (18)	31.19 ± 11.05	28 (20)	−11.26 ± 9.34	−12 (14)	−4.27	< 0.001
	Con. (*n =* 33)	42.03 ± 10.26	43 (16)	41.48 ± 10.65	42 (12)	−0.55 ± 10.64	1 (16)		
QoL	Exp. (*n =* 31)	0.82 ± 0.13	0.82 (0.15)	0.89 ± 0.12	0.91 (0.13)	0.07 ± 0.12	0.09 (0.14)	1.84	0.066
	Con. (*n =* 33)	0.78 ± 0.15	0.77 (0.25)	0.81 ± 0.11	0.77 (0.13)	0.03 ± 0.10	0 (0.96)		
Mobility	Exp.	2.48 ± 0.51	2 (1)	2.84 ± 0.37	3 (0)	0.35 ± 0.49	0 (1)	3.21	< 0.001
	Con.	2.52 ± 0.51	3 (1)	2.48 ± 0.51	2 (1)	−0.03 ± 0.39	0 (0)		
Self–Care	Exp.	2.87 ± 0.43	3 (0)	2.94 ± 0.25	3 (0)	0.06 ± 0.36	0 (0)	1.03	0.302
	Con.	2.91 ± 0.29	3 (0)	2.91 ± 0.29	3 (0)	0.00 ± 0.00	0 (0)		
Usual activities	Exp.	2.81 ± 0.40	3 (0)	3.00 ± 0.00	3 (0)	0.19 ± 0.40	0 (0)	1.02	0.31
	Con.	2.61 ± 0.50	3 (1)	2.70 ± 0.47	3 (1)	0.09 ± 0.38	0 (0)		
Pain/discomfort	Exp.	2.16 ± 0.58	2 (1)	2.29 ± 0.59	2 (1)	0.13 ± 0.67	0 (1)	−0.63	0.53
	Con.	1.88 ± 0.65	2 (1)	2.12 ± 0.55	2 (0)	0.24 ± 0.50	0 (1)		
Anxiety/depression	Exp.	2.52 ± 0.57	3 (1)	2.74 ± 0.58	3 (0)	0.23 ± 0.56	0 (1)	1.5	0.064
	Con.	2.45 ± 0.56	2 (1)	2.42 ± 0.61	2 (1)	−0.03 ± 0.53	0 (0)		

Con., Control group; Exp., Experimental group; QoL, Quality of life; M, mean; SD, Standard deviation.

## 4. Discussion

The results showed that the cognitive function scores improved, depression and loneliness scores decreased, and the difference between groups in the pre-post change was statistically significant. In contrast, the QoL score improved, but the between-group difference was not statistically significant.

In this study, the cognitive function score of the experimental group increased compared to that before the intervention, while that of the control group decreased. Regarding the MMSE tool, a decrease of 1–3 points can be judged as a clinically meaningful result (29). Therefore, it can be concluded that the program using the PIO robot improved the cognitive function of older adults living alone. There have been few studies on cognitive intervention programs using social robots for older people, and most of them have shown an improvement in cognitive function scores, but without any statistically significant difference (16, 30–33). In particular, the Sil-Bot robot was not able to derive statistically significant results from cognitive function variables even though it was composed of a program including the elements of CACR; however, this program was distinct as it derived statistically significant results. In a study that evaluated cognitive function using the harp-seal PARO robot for older people with dementia, the cognitive function score decreased after the intervention (18, 20, 34). This seems to be because the robot intervention consisting of an AAT program is hard to operate among older adults who have not been diagnosed with dementia; it is difficult to proceed because the program is monotonous, and its effectiveness is difficult to verify in older people with dementia. Multitasking ability demands, such as allocating and shifting attention, may induce additional brain activity in older adults (16). This program required a lot of multitasking between the PIO and participants, and the session was configured to increase emotional interaction with the PIO. A study showed that a multi-domain cognitive training program is more suitable for increasing brain neuroplasticity than monotherapy such as occupational therapy, exercise therapy, or art therapy (35); moreover, repetition was shown to enhance cognitive function in older adults, which is consistent with previous studies showing that it is effective to apply a program that integrates emotional and physical stimulation as well as cognitive stimulation (36).

The experimental group participating in the program had a more significant decrease in depression scores than the control group. This is also consistent with the results of a previous study that simultaneously confirmed the subject's cognitive function and depression, using the Sil-Bot robot (31). However, Oh et al. (37) configured a complex program with play content including recreational functions preferred by older people as a Silver-Care-Robot so that the robot and participants could interact, but both cognitive function and depressive variables decreased after the intervention. Petersen et al. (18) and Liang et al. (34), who performed interventions with the PARO robot for older adults with dementia, reported a statistically significant decrease in depression, but Robinson et al. (38) and Júranson et al. (39) found that depression scores in the experimental group increased after the intervention. As such, depending on the type of social robot and characteristics of the intervention method, the effect on the depressive variable is inconsistent. Social robots are useful for their attachment to humans, simple communication, and responsiveness to learning and training, such as the relationship between humans and dogs (40). Based on the storytelling method for participants to grow their PIO, this program was designed to increase attachment through interactions between participants and the PIO. By giving the PIO a name, making clothes, and so on, it was possible to nurture the PIO and encourage the participants to perform the program well, and by performing various actions, emotional communion with the participant was increased. Therefore, it is judged that this program increased the attachment between participants and the PIO and lowered depression. This study was conducted during the COVID-19 pandemic, and conversations between participants were prohibited during the program to ensure their safety. Therefore, the study results can be seen as the interaction effect between the participant and the PIO, rather than the interaction effect between the participants during the program.

In this study, the experimental group had a more statistically significant decrease in loneliness than the control. It was judged as effective to have an emotional bond through attachment in the process of hatching and growing from an egg, just like raising a real pet to promote an emotional bond between the participant and PIO. In a previous study that used the dog-shaped social robot AIBO, Kanamori et al. (41) reported that loneliness in older people decreased, but this was not statistically significant; Banks et al. (42) reported that loneliness decreased statistically significantly. It was reported that loneliness decreased in the intervention using the PARO robot, but the difference was not statistically significant (38). Loneliness variables were inconsistent in the social robot interventions.

Loneliness is a subjective emotion that appears by recognizing a lack of close social contact or emotional bonding (43); depression and loneliness closely interact with each other (44). This study was conducted during the COVID-19 pandemic by minimizing interaction and social contact between participants to confirm the significant effect of depressive variables. Therefore, to reduce depression or loneliness, it is necessary to construct a program for social contact and emotional bonds between subjects through social robots, or to develop a program for creating emotional bonds between social robots and participants. Older people who regularly participate in exercise programs for physical activity have improved self-esteem and reduced loneliness (40, 45). This program consisted of “Gymnastics with PIO” and participants continued stretching before and during each session. Participants observed that they paid more attention to the movement of the PIO than to the movie clip on the tablet PC.

The quality of the QoL score improved in the experimental group, but the difference was not statistically significant. Similarly, Júranson et al. (46) reported that the PARO robot improved the QoL score for older people with moderate dementia, but it was not statistically significant; however, the score was statistically significant for those with severe dementia. Valentï Soler et al. (20) reported that QoL was significantly improved by using the PARO robot. The QoL measurement tool used in this study was EQ-5D-3L. According to a study on the validity and reliability of the EQ-5D tool targeting Koreans, Koreans do not actively express their social and cultural health problems, showing a relatively high ceiling effect (47). Therefore, the participants answered their health-related questions positively in the pre-measurement; however, in the post-measurement, they may have answered their health status more exaggeratedly, so it was judged that the QoL score decreased after the intervention. Therefore, after assessing the items in the instrument and the composition of this program, it is recommended that subsequent researchers use the EQ-VAS (Euro Quality of life-Visual Analog Scale) rather than the EQ-5D-3L.

## 5. Limitations

This study was conducted during the COVID-19 pandemic. Although it was implemented as a group program, social interactions between participants were controlled for their safety. Therefore, it was possible to control for confounding variables through interactions between participants. Randomized controlled trials could not be conducted because of restrictions on gatherings due to COVID-19 and the anxiety of older adults living alone or within group programs. In addition, pre-and follow-up measurements are required to confirm whether the effect of the program continues; however, follow-up measurements were not performed.

## 6. Conclusions

The PIO robot program was confirmed to be effective in cognitive function, depression, and loneliness for the older adults living alone. If this program is applied to older adults living alone in the community, cognitive decline and progression to dementia may be prevented. This reduce the nation's social burden. When nurses directly implement the PIO program intervention, they can provide customized nursing care by identifying the actual function of the patient, which can be utilized effectively in nursing practice.

## Data availability statement

The raw data supporting the conclusions of this article will be made available by the authors, without undue reservation.

## Ethics statement

This study was approved by the Institutional Review Board (IRB) of Seoul National University. The approval number is IRB No. 2108/002-012. The patients/participants provided their written informed consent to participate in this study.

## Author contributions

The author confirms being the sole contributor of this work and has approved it for publication.
